# On-line Smoothing and Error Modelling for Integration of GNSS and Visual Odometry

**DOI:** 10.3390/s19235259

**Published:** 2019-11-29

**Authors:** Thanh Trung Duong, Kai-Wei Chiang, Dinh Thuan Le

**Affiliations:** 1 Department of Geomatics and Land-administration, Hanoi University of Mining and Geology, Hanoi 122000, Vietnam; duong_trung2004@yahoo.com; 2 Department of Geomatics, National Cheng Kung University, Tainan 701, Taiwan; kwchiang@mail.ncku.edu.tw

**Keywords:** GNSS, INS, integration, navigation, visual odometry, on-line smoothing, error modelling

## Abstract

Global navigation satellite systems (GNSSs) are commonly used for navigation and mapping applications. However, in GNSS-hostile environments, where the GNSS signal is noisy or blocked, the navigation information provided by a GNSS is inaccurate or unavailable. To overcome these issues, this study proposed a real-time visual odometry (VO)/GNSS integrated navigation system. An on-line smoothing method based on the extended Kalman filter (EKF) and the Rauch-Tung-Striebel (RTS) smoother was proposed. VO error modelling was also proposed to estimate the VO error and compensate the incoming measurements. Field tests were performed in various GNSS-hostile environments, including under a tree canopy and an urban area. An analysis of the test results indicates that with the EKF used for data fusion, the root-mean-square error (RMSE) of the three-dimensional position is about 80 times lower than that of the VO-only solution. The on-line smoothing and error modelling made the results more accurate, allowing seamless on-line navigation information. The efficiency of the proposed methods in terms of cost and accuracy compared to the conventional inertial navigation system (INS)/GNSS integrated system was demonstrated.

## 1. Introduction

Global navigation satellite systems (GNSSs) are commonly applied for positioning and navigation. The positioning solution is accurate and continuous if direct signals from more than four satellites are received. However, in GNSS-hostile environments, where signals are reflected and blocked, the availability and accuracy of GNSS-based vehicle navigation systems are degraded significantly [1]. To overcome this issue, the integration of a GNSS and another navigation system has been proposed. The most common integration is a GNSS and an inertial navigation system (INS). Even though an INS/GNSS integrated system can improve navigation capability in GNSS-denied environments, it depends deeply on the cost of used inertial sensors and the outages in GNSS signals [2]. Inertial systems of a tactical-grade or higher can compensate to obtain appropriate positioning, accuracy, and sustainability during long-term GNSS signal unavailability [3]. For instance, in the case of a GNSS outage lasting one minute, systems with high-grade inertial sensors can achieve a real-time positioning accuracy of less than three meters. Nevertheless, the use of these sophisticated inertial sensors is limited for applications such as the primary navigation module for general land vehicles due to their price and government regulation [1,2]. Low-cost microelectromechanical systems’ (MEMS) inertial sensors are thus applied as a potential complementary component. However, the positioning error of integrated systems drifts quickly when GNSS signals are blocked due to the poor performance of these inertial sensors.

Visual odometry (VO) using a camera is an alternative or supplemental navigation solution in GNSS-hostile environments [4,5]. VO estimates the ego-motion of an agent given consecutive images captured by one or more cameras. The output of VO is the relative translation and rotation of the carrier platform in the initial camera frame. Compared with wheel odometry, VO is not affected by wheel slip in uneven terrain or other adverse conditions [6]. VO utilizes low-cost sensors and the frames captured by a camera can provide a large amount of information that can be used for different purposes, including navigation [7,8]. However, its performance depends on the illumination of the environment, the texture of the static scene, and the overlap between consecutive frames [6]. The present study integrated VO and GNSS to utilize the advantages and overcome the limitations of each system in stand-alone mode.

According to the literature, the integration of VO and GNSS has been investigated. Dusha and Mejias [9] introduced a loosely coupled global positioning system (GPS)/VO integration. Their method was demonstrated using numerical simulations and was evaluated using real flight data. However, they mainly focused on the observability properties of the GPS/VO filtering instead of optimal estimation or real-time navigation performance. Moreover, in real experiments, they used a downward-looking camera, which is different in the context of the forward-facing camera’s popular application in navigation. Schreiber, Königshof [10] presented a method for integrating GNSS measurements from a low-cost receiver with a locally accurate visual odometry obtained from an on-board low-cost stereo camera system. Although their system had robust localization, the achieved accuracy was insufficient for autonomous driving. Chen, Hu [11] investigated the integration of measurements from a low-cost GNSS and monocular camera measurements in a simultaneous localization and mapping (SLAM) system. The proposed system can perform in real time and achieve the absolute position, attitude, and metric scale of the vehicle. However, this system is mainly based on an ORB-SLAM framework with optimization-based algorithms, so it is unable to apply smoother in order to improve the accuracy of navigation performance. The scale is initialized in the beginning instead of being included in the state for estimation. Furthermore, they experimented with an available KITTI dataset, which ORB-SLAM system has been successful working with, instead of real experiments with their own configurations. 

In this research, we proposed a real-time VO/GNSS integrated navigation system that utilizes on-line smoothing based on the extended Kalman filter (EKF) and the Rauch-Tung-Striebel (RTS) smoother. VO error modelling was also proposed to estimate the VO error and compensate the incoming measurements. The contributions addressed herein are, firstly, that the result of this work contributes to confirm the advancement of VO/GNSS integration, which can be compared to a conventional INS/GNSS approach. Secondly, on-line smoothing and error modelling were applied to enhance the performance that makes the system capable of robust ground vehicle navigation. Finally, the integrated system was validated through two live sets of data collected in various GNSS-hostile environments (e.g., under a tree canopy, urban area), together with a centimeter-accurate reference system, to demonstrate performance of the proposed system.

The remainder of this paper is organized as follows: Section 2 provides an overview of VO. Section 3 presents the design of the integrated architecture, the system model, and the measurement model. Section 4 describes data fusion strategies. The experimental results and discussion are presented in Section 5. Finally, some concluding remarks and a brief outline for future research are presented in Section 6.

## 2. Visual Odometry

### 2.1. General Concept of VO

**Figure 1:**
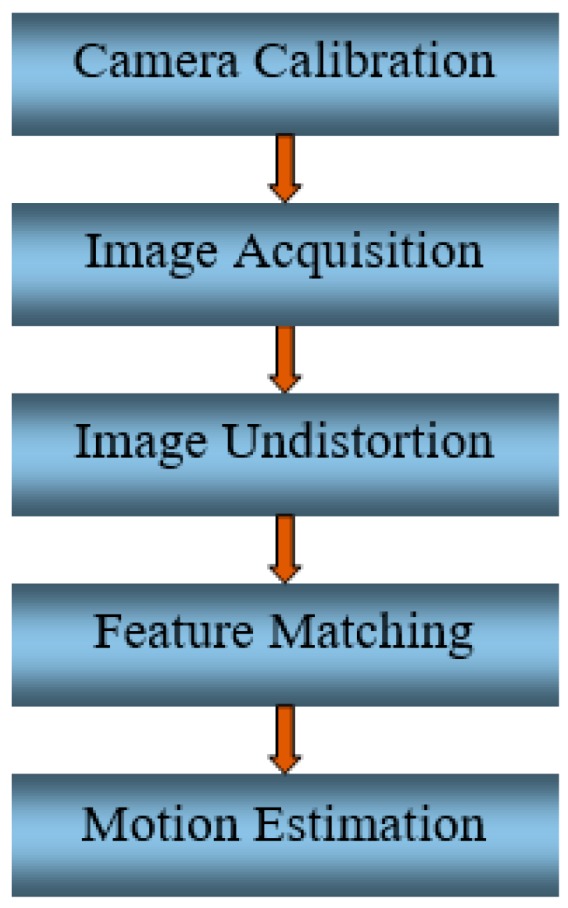
Flowchart of visual odometry (VO).

VO can be divided into monocular or stereo VO. Monocular VO uses a single camera to derive ego-motion based on feature matching (or feature tracking) between consecutive images, whereas stereo VO uses a pair of cameras. Compared to monocular VO, stereo VO is more accurate but has a higher computational burden [12]. Therefore, monocular VO is the first choice for real-time applications [13]. The flowchart of VO is described in Figure 1.

### 2.2. Camera Calibration

Camera calibration is used to determine the camera’s intrinsic and distortion parameters. The camera’s intrinsic parameters are usually presented in the form of a matrix that includes the camera’s focal length and principle point. The distortion parameters are usually expressed in terms of the tangential distortion and the radial distortion coefficients of the lenses. The camera calibration can be implemented using commercial software or free tools such as Bouget’s Matlab Camera Calibration Toolbox [14] or OpenCV [15]. Several calibration processes have been proposed [16,17]. In this research, OpenCV, with a checkerboard pattern, was used for camera calibration.

### 2.3. Image Acquisition and Undistortion

A sequence of images is obtained from the camera at a certain frame rate. For real-time applications, the frame rate is critical. It should be small enough to implement in real time, but large enough to have an overlap for deriving a VO solution. The frame rate is adjusted depending on the size of the image and the moving speed of the vehicle. For example, for an image size of 808 × 608 pixels and an average vehicle speed of 2 m/s, the frame rate should be 3 frames per second for real-time applications [18].

**Figure 2:**
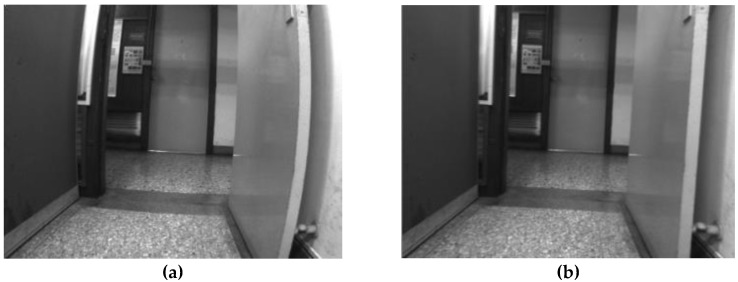
(**a**) Distorted image and (**b**) its correction.

The camera lens can distort images. Objects in distorted images look different from the way they do in reality (e.g., straight lines become curved). The magnitude of distortion increases from the center to the edges of images and varies with viewpoint. Based on the distortion parameters determined by camera calibration, a distorted image can be corrected to improve the performance of subsequent image processing. Figure 2 compares a distorted image and its correction.

### 2.4. Feature Matching

**Figure 3:**
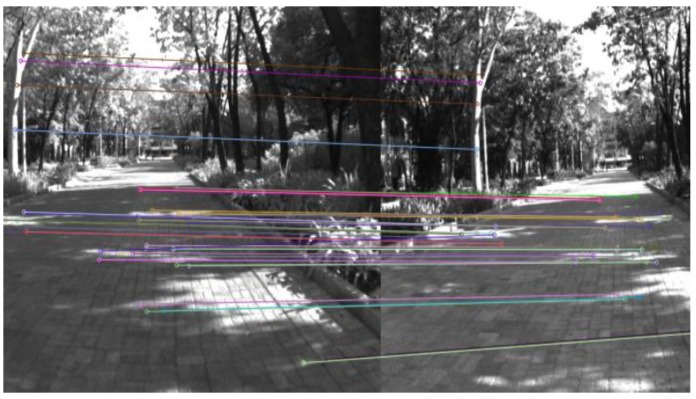
Illustration of feature matching.

To derive a VO solution based on a feature-based method, feature points between consecutive images must be matched or tracked. This process commonly has two stages. In the first stage, feature detection is used to find key points that are the most suitable for matching to features in other images [19]. Various feature detection algorithms have been proposed [20]. According to Fraundorfer and Scaramuzza [20], point-feature detectors can be divided into two groups, namely corner detectors and blob detectors. The representatives for corner detectors are FATS, Harris, Shi-Tomasi, Moravec, and Forstner, whereas SIFT, SURF, and CENSURE are typical algorithms for blob detectors. Each algorithm has its own advantages and disadvantages. In general, blob detectors are more distinctive and better localized in scale, but corner detectors are fast to compute and are better localized in image position. In the second stage, the corresponding feature is looked for in the subsequent images. This process is called feature matching or tracking. In this research, the SIFT [21] feature matching algorithm was applied. Most of the SIFT algorithm’s power lies in its robust descriptor, which is stable against changes in illumination, rotation, and scale. Figure 3 illustrates the feature matching of two images.

### 2.5. Motion Estimation

**Figure 4:**
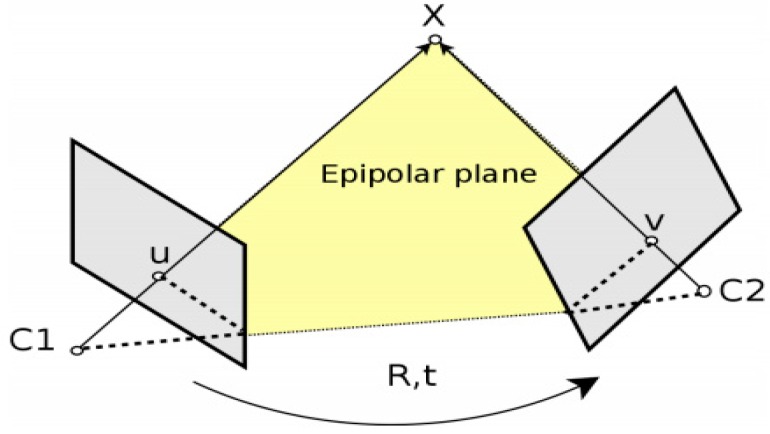
Principle of epipolar constraint in VO.

Motion estimation is used to determine camera transformation between the current image and the previous image. The mathematical principle of motion estimation is based on an epipolar constraint [6]. In Figure 4, a camera undergoes motion from C1 to C2 with rotation R and translation t. Given a three-dimensional (3D) point X, the projection of X in the image plane at C1 is u and the corresponding image point at C2 is ν. The epipolar constraint equation is formed as

(1) $$u^{T}Ev=0$$

where E = [t] × R is essential matrix.

Equation (1) can be rewritten in the form:

(2) Ae=0

where A represents the components u and v, and e represents the components of E.

To solve Equation (2), eight point correspondences are normally required [22]. Fewer point correspondences are required if additional constrains are used in the motion condition [6,23]. Equation (2) is solved based on the principle of minimizing the projection error to determine R and t.

(3) Argmin‖Ae‖

Note that Equation (2) is satisfied with static points. However, a real scenario may contain a moving object and erroneous conditions; thus, outlier removal is necessary. The RANSAC algorithm is commonly used for this task [24]. After outlier removal using RANSAC, the number of inliers is determined. A larger number of inliers usually leads to a more reliable solution in motion estimation. Therefore, in this research, a number of inliers were used to build the error model of VO.

Equation (2), with condition (3), is solved utilizing singular value decomposition (SVD) to determine the essential matrix E. Rotation matrix R and translation vector t are then determined [23].

Let E = U∑V^T^ be the SVD of E. Then,

(4) $${\left[t\right]}_{\times }=VW\Sigma V^{T}$$

(5) $$R=UW^{T}V^{T}$$

(6) $$W=\left[\begin{array}{ccc} 0 & 1 & 0\\ 1 & 0 & 0\\ 0 & 0 & 1 \end{array}\right]$$

where [t]× is the skew matrix of the translation vector t [25].

Given the determined translation vector t and the rotation matrix R, the transformation of the camera at time *k* is formed as

(7) $$T_{k}=\left[\begin{array}{cc} R_{p} & t_{p}\\ 0 & 1 \end{array}\right]$$

Then, the pose of the camera at time *k* can be determined using a concatenated equation:

(8) $$C_{k}=C_{k-1}T$$

where *C_k_* and *C*_*k*−1_ are the poses of the camera at times *k* and *k* − 1, respectively. The solution of VO is the pose of the camera, expressed in the initial camera frame. Its error will accumulate over time if no external constraint is applied.

## 3. Integration Architecture

### 3.1. General Architecture Design

**Figure 5:**
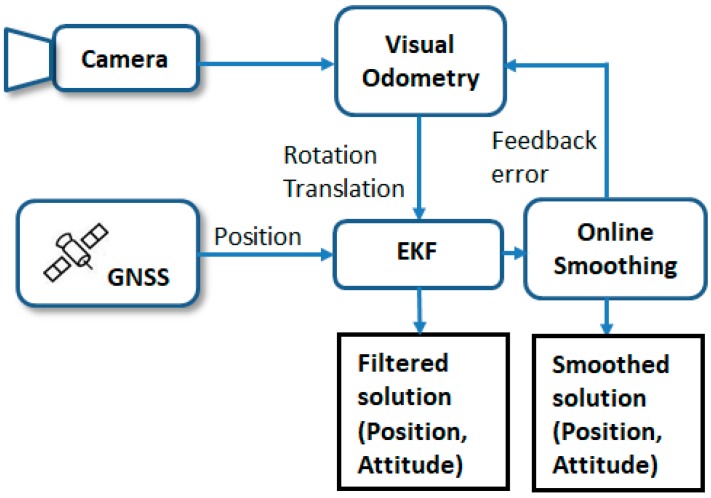
Proposed VO/GNSS integration scheme.

In this system, a loosely coupled scheme for VO/GNSS integration was designed. The images taken by the camera were processed by a VO mechanization to derive the translation and rotation in camera frame. GNSS provides absolute position as the major measurement update. An EKF was designed for multi-sensor data fusion and an RTS smoother was applied to provide more accurate navigation solutions. The integration scheme is shown in Figure 5.

### 3.2. Model Design

System and measurement models are needed for fusing data with an estimation tool such as the EKF [1,3]. In this research, the principle ego-motion of the VO was utilized to create a system model for the EKF. The measurements from GNSS were used to form measurement models.

The system model was created by error analysis utilizing perturbation methods of VO. The details of the derivation can be found in the study of Dusha and Mejias [9]. The time-continuous VO error model is formed as

(9) $$\begin{array}{ll} \left[\begin{array}{c} \delta \dot{\lambda }\\ \delta {\dot{r}}^{n}\\ \dot{\psi } \end{array}\right] & =[\begin{array}{ccc} 0 & 0_{1\times 3} & 0_{1\times 3}\\ {\tilde{R}}_{b}^{n}{\tilde{T}}^{b} & 0_{3\times 3} & -\tilde{\lambda }{\left[{\tilde{R}}_{b}^{n}{\tilde{T}}^{b}\right]}_{\times }\\ 0_{3\times 1} & 0_{3\times 3} & 0_{3\times 3} \end{array}]\left[\begin{array}{c} \delta \lambda \\ \delta r^{n}\\ \psi \end{array}\right]\\ & +\left[\begin{array}{ccc} 1 & 0_{1\times 3} & 0_{1\times 3}\\ 0_{3\times 1} & \tilde{\lambda }{\tilde{R}}_{b}^{n} & 0_{3\times 3}\\ 0_{3\times 1} & 0_{3\times 3} & {\tilde{R}}_{b}^{n} \end{array}\right]\left[\begin{array}{c} v\\ \delta {\tilde{T}}^{b}\\ \delta \omega_{nb}^{b} \end{array}\right] \end{array}$$

where $\delta \dot{\lambda }$, $\delta {\dot{r}}^{n}$, and $\dot{\psi }$ are continuous-time derivatives of length scale factor, position, and attitude in local-level frame (n-frame), respectively. ${\tilde{R}}_{b}^{n}$ is the estimated rotation matrix from the body frame (b-frame) to the n-frame. ${\tilde{T}}^{b}$ is an estimated translation expressed in the b-frame. $\delta {\tilde{T}}^{b}$ and $\delta \omega_{nb}^{b}$ are translation and rotation errors in the b-frame, respectively.

Equation (9) can be presented in continuous-time system model:

(10) $$\dot{x}=Fx+Gu$$

Equation (10) can be transformed into a discrete-time form [1,26]:

(11) $$x_{k+1}=\Phi_{k}x_{k}+w_{k}$$

where $x_{k}={\left[\delta \lambda \;\delta r^{n}\;\psi \right]}_{7\times 1}^{T}$ is the state vector at time (epoch) *k*, $\Phi_{k}$ is the discrete-time transition matrix from epoch *k* to epoch *k* + 1, and $w_{k}$ is process noise [27,28].

Measurement model for the EKF is expressed as

(12) $$z_{k}=Hx_{k}+v_{k}$$

where *H* is the design matrix or geometry matrix, $z_{k}$ and $v_{k}$ are measurement and its noise, respectively.

For positional measurement provided by GNSS, the measurement model for the EKF is formed as

(13) $$z=r_{VO}^{e}-r_{GPS}^{e}=HR_{e}^{n}x_{k}+\varepsilon_{r}$$

where $r_{VO}^{e}$ and $r_{GPS}^{e}$ are the positional vectors provided by VO and GNSS in the Earth-centered Earth-fixed frame (e-frame), respectively, and $R_{e}^{n}$ is the rotation matrix from the e-frame to the n-frame.

(14) $$H=\left[\begin{array}{ccc} 0 & I_{1\times 3} & O_{1\times 3} \end{array}\right]$$

*H* is a measurement-mapping matrix describing the relationship between the measurement vector and the state vector. $\varepsilon_{r}$ is the position noise of GNSS measurements.

## 4. Data Fusion Strategies

### 4.1. Estimation with Extended Kalman Filter

EKF equations are divided into the following two groups: time prediction and measurement update. The time prediction equations convert state and covariance from the current epoch state (*k*) to the next epoch (*k* + 1) [27].

(15) $${\hat{x}}_{k+1}^{-}=\Phi_{k}{\hat{x}}_{k}^{+}$$

(16) $$P_{k+1}^{-}=\Phi_{k}P_{k}^{+}\Phi_{k}^{T}+Q_{k}$$

where (ˆ) denotes estimation and (-) and (+) denote the estimated values after prediction and update, respectively.

When GNSS measurements are observed, the following measurement update equations are activated:

(17) $$K_{k}=P_{k}^{-}H_{k}^{T}{\left(H_{k}P_{k}^{-}H_{k}^{T}+R_{k}\right)}^{-1}$$

(18) $${\hat{x}}_{k}^{+}={\hat{x}}_{k}^{-}+K_{k}\left(z_{k}-H_{k}{\hat{x}}_{k}^{-}\right)$$

(19) $$P_{k}^{+}=\left(I-K_{k}H_{k}^{T}\right)P_{k}^{-}$$

where $K_{k}$ is the Kalman gain, $R_{k}$ is the covariance matrix of GNSS measurements. All noise terms are considered to be white with known covariance and uncorrelated with each other.

### 4.2. On-Line Smoothing

In this research, on-line smoothing was applied. The remaining time in each epoch was utilized to perform smoothing during operation time to increase the capability of the system in terms of accuracy. Following Chiang, Duong [29], on-line smoothing was originated from an RTS smoother algorithm. The principle of this algorithm is introduced below.

According to Rauch, Tung [30], smoothing is targeted to estimate probability density function (PDF) of the states based on all measurements from time *k* to time *N*, where *k* ≤ *N*:

(20) $$P\left(x_{k},x_{k+1}|z_{N}\right)=P\left(x_{k+1}|x_{k}\right)P\left(x_{k}|z_{k}\right)P\left(z_{k+1},\ldots ,z_{N}|x_{k+1}\right)P\left(z_{k}\right)$$

The RTS smoother finds optimal estimation by applying a maximum likelihood of state vectors based on aiding measurements vectors:

(21) $$maxL\left(x_{k},x_{k+1}|z_{N}\right)=maxlogP\left(x_{k},x_{k+1}|z_{N}\right)$$

where $L\left(x_{k},x_{k+1}|z_{N}\right)$ is the likelihood of $x_{k},x_{k+1}$ based on $z_{N}$.

The estimated and covariance of states are achieved by resolving criteria in Equation (21):

(22) $${\hat{x}}_{k|N}={\hat{x}}_{k}+C_{k}\left({\hat{x}}_{k+1|N}-{\hat{x}}_{k+1}\right)$$

(23) $$P_{k|N}=P_{k}+C_{k}\left(P_{k+1|N}-P_{k+1}\right)C_{k}^{T}$$

where ${\hat{x}}_{k|N}$ and $P_{k|N}$ are smoothed states and covariance at time k based on information up to time *N* (*k* ≤ *N*), respectively, ${\hat{x}}_{k}$ and $P_{k}$ are estimated states and covariance provided by the EKF at time *k*, respectively, and $C_{k}$ is cross covariance, calculated in the following equation:

(24) $$C_{k}=P_{k}\Phi_{k,k+1}^{T}P_{k+1}^{-1}$$

**Figure 6:**
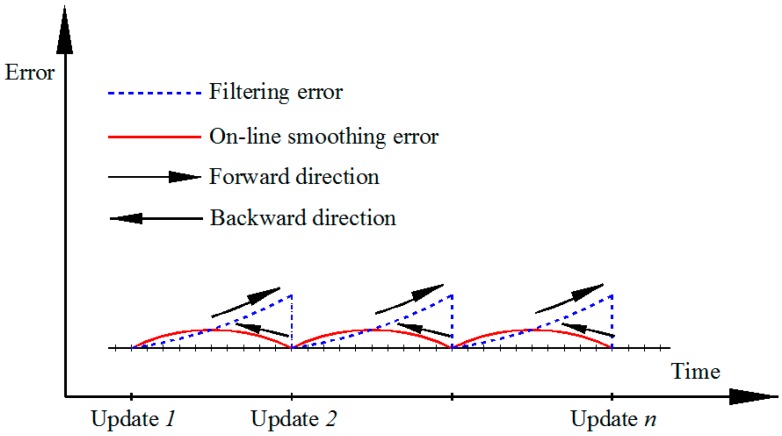
Error illustration of on-line smoothing.

**Figure 7:**
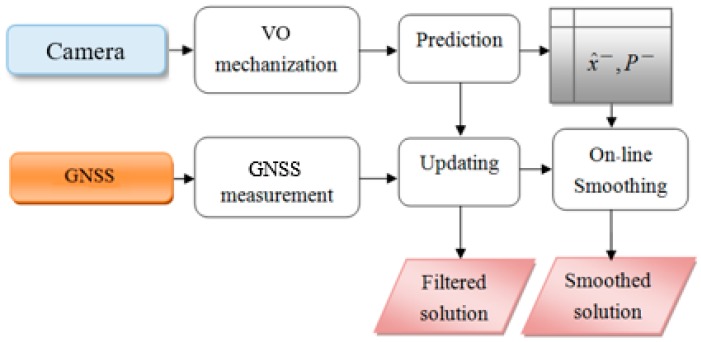
Flowchart of filtering and on-line smoothing.

With on-line smoothing, the processing scheme can be done with real-time data. Figure 6 indicates the processing principle and error performance of on-line smoothing. The integrated scheme with on-line smoothing of VO/GNSS is described in Figure 7.

### 4.3. Error Modelling with On-Line Smoothing

Error modelling in VO/GNSS integration includes VO noise modelling, the length scale factor, and heading drift. It is assumed that the estimates from the smoother are better in quality and provide more output solutions compared to the EKF; therefore, the output from the smoothing solutions in each smoothing window is used for modelling error.

(25) $$Q_{k}=\left[\varepsilon \varepsilon^{T}\right]$$

(26) $$\varepsilon_{k}=x_{k|N}-{\hat{x}}_{k}^{-}$$

where $x_{k|N}$ is the smoothing solution and ${\hat{x}}_{k}^{-}$ is the prediction of the EKF.

$Q_{k}$ is the system error model at time *k*. It is used as the error model for the next estimation step at time *k* + 1.

With this scheme, the system error model is updated every updating step of the EKF whenever the GNSS measurement is available.

In the VO/GNSS integration, the position and the length scale factor of VO are updated continuously based on GNSS data. However, it is no longer updated because GNSS does not provide an attitude parameter. Consequently, the position of the system drifts quickly during GNSS outages due to the drift of the heading, especially during turning. Thus, heading error modelling is used to estimate the heading error based on on-line smoothing as

(27) $$∆h_{k}=mean\left(h_{k|N}-h_{k}^{-}\right)$$

where $h_{k|N}$ is the smoothed heading and $h_{k}^{-}$ is the predicted heading by the EKF at time *k*. The heading at time *k* + 1 is then estimated.

(28) $$h_{k+1}=h_{k}+∆h_{k}/2$$

## 5. Experiment and Discussion

The testing system comprised a monoband camera (Blackfly, Point Grey) with a resolution of 808 × 608 (0.5 MP). GNSS data were provided by a double-frequency GNSS receiver (ProPak V3, NovAtel). The original GNSS output data rate was 1 Hz (one data record per second); however, for testing, the GNSS data rate was decreased to 0.05 Hz (one data record every 20 s).

**Figure 8:**
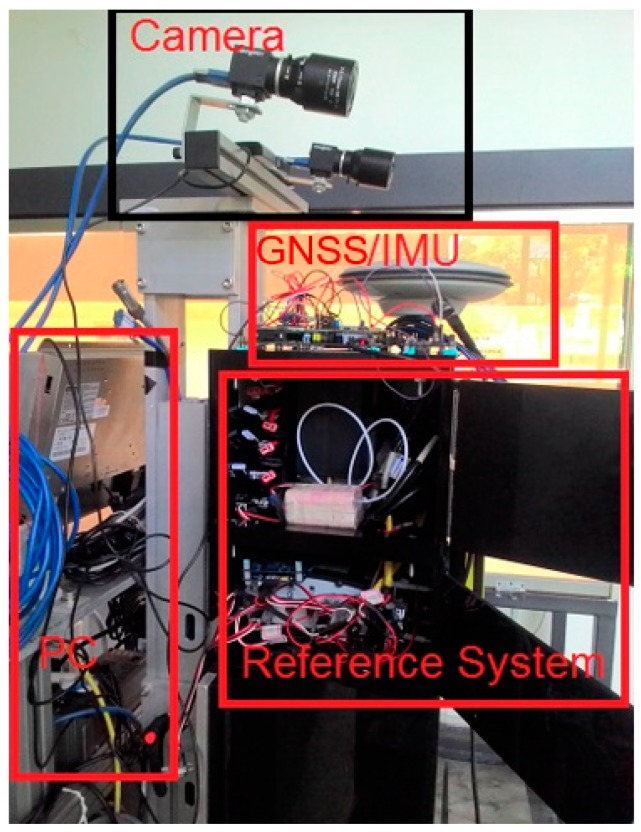
Testing platform.

The system for generating reference composed a medium tactical-grade inertial measurement unit (IMU) (C-MIGIT) and a dual-frequency geodetic-grade GNSS receiver (ProPak V3, NovAtel). Some additional ground control points in the GNSS-denied environment were included to guarantee that the reference solution was at the centimeter level of accuracy. The system was set up on a platform for testing, as shown in Figure 8. The reference trajectories were generated using tightly coupled integration with a smoothing algorithm using the commercial IMU/GNSS processing software, Inertial Explore. A testing software module was written and designed in C++ programming language to acquire and process data.

**Figure 9:**
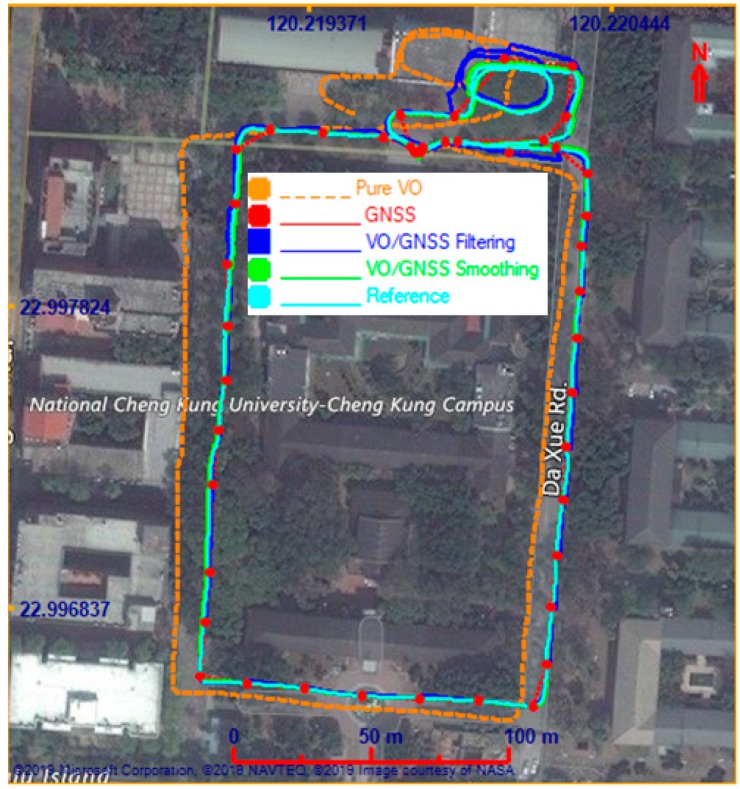
Positions of various solutions on the map.

**Figure 10:**
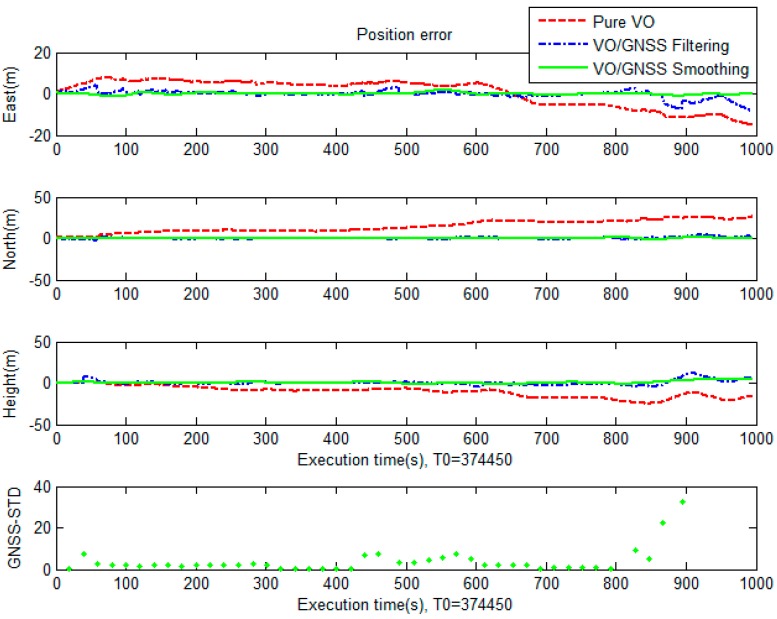
Graphical comparison of the positional error between various solutions.

In the first test, the testing field was carried out at a GNSS-hostile environment at a campus of National Cheng Kung University, Tainan, Taiwan. The testing trajectory is displayed in Figure 9. For performance evaluation, the solutions provided by pure VO, VO/GNSS using an original EKF, and VO/GNSS with on-line smoothing were analyzed. Figure 9 shows the positions of these solutions on the map and Figure 10 indicates a graphical comparison of the positional error between solutions. The numerical statistics in terms of the positional root-mean-square error (RMSE) are shown in Table 1.

It can be seen from the results that the positional error of the pure VO grows quickly over time. For VO/GNSS fusion using an EKF, the VO position is constrained by GNSS, and thus, its accuracy improves significantly (by 82.3%) compared to that of pure VO. Moreover, the integrated solution can provide seamless navigation even with GNSS outages. With on-line smoothing, the smoother is activated whenever an updating measurement is found. Smoothing is performed backward from current to previous updating time, utilizing data which stores in temporary dynamic arrays. The navigation solution with smoothing is more accurate (by 92.4%) than pure VO.

**Figure 11:**
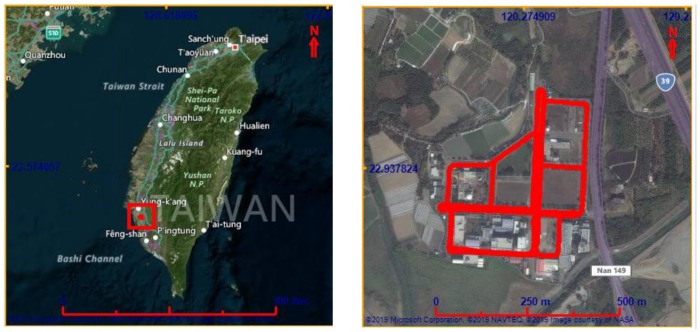
Second test scenario.

**Figure 12:**
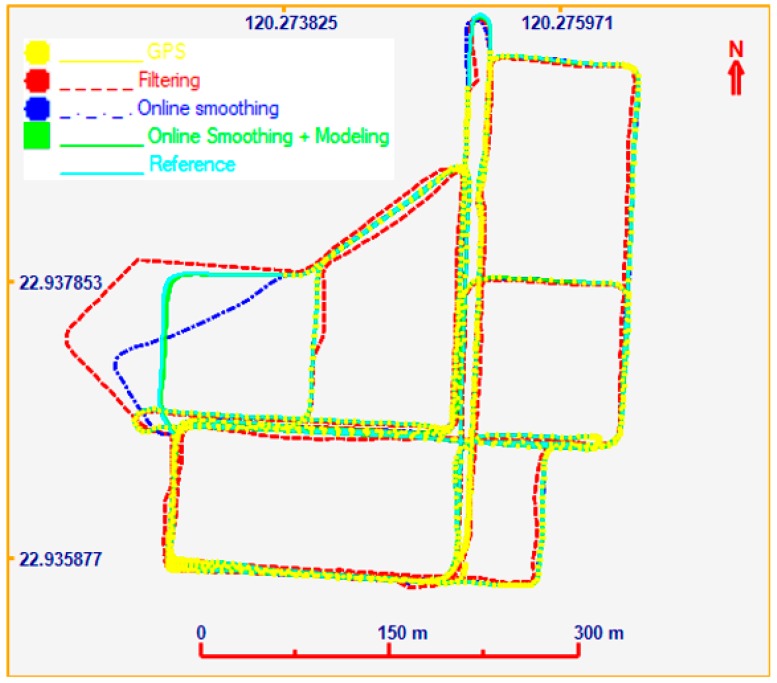
Ground trajectories.

**Figure 13:**
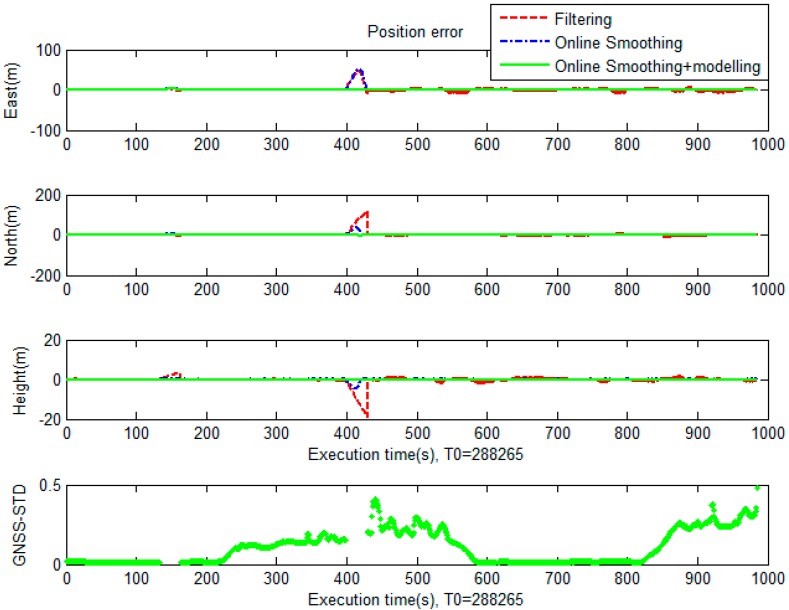
Graphical comparison of the positional error between solutions in the second test.

For the second test, the data set was collected at the Kuei-Jen Campus, National Cheng Kung University, where the GNSS satellite is good for evaluation, as show in Figure 11. In this test, the testing equipment was similar to that of the first test. Two simulated GNSS outages were generated. The performance analysis focuses on the VO/GNSS solution with an EKF, on-line smoothing, and on-line smoothing and error modelling. The comparison between the three solutions in terms of the ground trajectory and a graph are shown in Figure 12 and Figure 13, respectively. The numerical analysis results, in terms of the positional RMSE, are shown in Table 2.

According to the statistics in Table 2, the estimation accuracy in terms of position for smoothing is much better than that of the EKF. With on-line smoothing, the improvement in RMSE is about 50%. The heading error, however, still drifts over time, leading to a large position error, particularly during GNSS outages. For on-line smoothing and error modelling, the heading error was estimated; the accuracy improvement was 96.2% compared to that of the EKF.

## 6. Conclusions

This study proposed an integrated scheme of VO and GNSS with on-line smoothing and error modelling based on the EKF and the RTS smoother to overcome the issues of GNSS in GNSS-challenging environments and the problem of unbounded error in VO. A system that included a camera, a GNSS receiver, and an IMU was combined for testing and reference generation, and a console program written in C++ based on OpenCV was implemented to evaluate the proposed method. 

The testing results indicate that with an EKF used for data fusion, the RMSE of the 3D position is about 80 times lower than that of the VO-only solution. With on-line smoothing and error modelling, the predicted and updated information from the EKF were smoothed and the heading error was estimated. The results are thus more accurate and provide seamless on-line navigation information.

In feature-based approach, the static, salient, and repeatable features are tracked across the sequence images. Therefore, in future work, an algorithm that adopts outlier removal and more robust feature tracking to deal with complex environments (e.g., urban roads with many moving vehicles) will be developed. Non-linear, non-Gaussian filtering and a smoothing algorithm will be applied in the VO/GNSS integrated system to overcome the limitation of the EKF in terms of error modelling and highly dynamic movement.

## Figures and Tables

**Table 1:** Comparison of the positional root-mean-square error (RMSE) for the first test.

RMSE (m)	Pure VO	VO/GNSS EKF	VO/GNSS on-line Smoothing
**North**	6.689	2.054	0.522
**East**	15.601	1.195	0.525
**Up**	12.933	2.933	1.454
**3D**	21.34	3.775	1.632
**Improvement (%)**	-	82.3	92.4

**Table 2:** Comparison of the positional RMSE for the second test.

RMSE (m)	VO/GNSS EKF	On-line Smoothing	On-Line Smoothing and Error Modelling
**North**	5.606	5.994	0.255
**East**	12.535	3.612	0.339
**Up**	1.98	0.458	0.322
**3D**	13.874	7.013	0.533
**Improvement (%)**	-	49.5	96.2

## References

[1] Chiang K.-W., Duong T.T., Liao J.-K. (2013). The performance analysis of a real-time integrated INS/GPS vehicle navigation system with abnormal GPS measurement elimination. Sensors.

[2] Shin E.-H. (2001). Accuarcy Improvement of Low Cost INS/GPS for Land Applications. Master’s Thesis.

[3] Titterton D., Weston J. (2004). Strapdown Inertial Navigation Technology.

[4] Maimone M., Cheng Y., Matthies L. (2007). Two years of visual odometry on the mars exploration rovers. J. Field Robot..

[5] Nistér D., Naroditsky O., Bergen J. (2006). Visual odometry for ground vehicle applications. J. Field Robot..

[6] Scaramuzza D., Fraundorfer F. (2011). Visual odometry, Part 1. IEEE Robot. Autom. Mag..

[7] Aqel M.O., Marhaban M.H., Saripan M.I., Ismail N.B. (2016). Review of visual odometry: Types, approaches, challenges, and applications. SpringerPlus.

[8] Tardif J.-P., George M., Laverne M., Kelly A., Stentz A. A new approach to vision-aided inertial navigation in Intelligent Robots and Systems (IROS). Proceedings of the 2010 IEEE/RSJ International Conference on Intelligent Robots and Systems.

[9] Dusha D., Mejias L. (2012). Error analysis and attitude observability of a monocular GPS/visual odometry integrated navigation filter. Int. J. Robot. Res..

[10] Schreiber M., Königshof H., Hellmund A.M., Stiller C. Vehicle localization with tightly coupled GNSS and visual odometry. Proceedings of the 2016 IEEE Intelligent Vehicles Symposium (IV).

[11] Chen X., Hu W., Zhang L., Shi Z., Li M. (2018). Integration of low-cost gnss and monocular cameras for simultaneous localization and mapping. Sensors.

[12] Howard A. Real-time stereo visual odometry for autonomous ground vehicles. Proceedings of the IROS 2008. IEEE/RSJ International Conference in Intelligent Robots and Systems 2008.

[13] Yousif K., Bab-Hadiashar A., Hoseinnezhad R. (2015). An overview to visual odometry and visual SLAM: Applications to mobile robotics. Intell. Ind. Syst..

[14] Bouguet J.-Y. Matlab Camera Calibration Toolbox.

[15] Bradski G., Kaehler A. Dr. (2000). Dobb’s journal of software tools. OpenCV Libr..

[16] Zhang Z. (2000). A flexible new technique for camera calibration. IEEE Trans. Pattern Anal. and Mach. Intell..

[17] Geiger A., Moosmann F., Car Ö., Schuster B. Automatic camera and range sensor calibration using a single shot. Proceedings of the 2012 IEEE International Conference in Robotics and Automation (ICRA).

[18] Zhang J., Singh S. (2018). Laser–visual–inertial odometry and mapping with high robustness and low drift. J. Field Robot..

[19] Chakraborty M. Feature Descriptor for Performing Visual Odometry. Proceedings of the International Conference on Engineering and Technology.

[20] Fraundorfer F., Scaramuzza D. (2012). Visual odometry, Part 2. IEEE Robot. Autom. Mag..

[21] Lowe D.G. (2004). Distinctive image features from scale-invariant keypoints. Int. J. Comput. Vis..

[22] Longuet-Higgins H.C. (1981). A computer algorithm for reconstructing a scene from two projections. Nature.

[23] Nistér D. (2004). An efficient solution to the five-point relative pose problem. IEEE Trans. Pattern Anal. Mach. Intell..

[24] Fischler M.A., Bolles R.C. (1981). Random sample consensus: A paradigm for model fitting with applications to image analysis and automated cartography. Commun. ACM.

[25] Jian Y.-D., Chen C.-S. (2010). Two-view motion segmentation with model selection and outlier removal by ransac-enhanced dirichlet process mixture models. Int. J. Comput. Vis..

[26] Shin E.-H. (2005). Estimation Techniques for Low-Cost Inertial Navigation, in UCGE Report. Ph.D. Dissertation.

[27] Gelb A. (1974). Applied Optimal Estimation.

[28] Brown R.G., Hwang P.Y. (1992). Introduction to Random Signals and Applied Kalman Filtering.

[29] Chiang K.-W., Duong T., Liao J.K., Lai Y.C., Chang C.C., Cai J.M., Huang S.C. (2012). On-line smoothing for an integrated navigation system with low-cost MEMS inertial sensors. Sensors.

[30] Rauch H.E., Tung F., Striebel C. (1965). Maximum likelihood estimates of linear dynamic systems. AIAA J..

